# Diet in Disease

**Published:** 1893-04-22

**Authors:** Ernest Hart

**Affiliations:** Bachelier-és-Sciences-es-Lettres (réstreint), formerly Student of the Faculty of Medicine of Paris, and of the London School of Medicine for Women


### Diet in Disease.

By Mrs. Ernest Hart, Bachelier-ès-Sciences-es-Lettres (rêstreint), formerly Student of the Faculty of Medicine of Paris, and of the London School of Medicine for Women.

### XX.—UNDER-FEEDING AND OVER-EATING.

Thanks to the careful and lengthened studies and the recorded observations of physicians and sanitarians, more especially of prison and army doctors, there is no subject in physiology on which we possess such accurate information as the amount and kind of food to be taken in proportion to the amount of work to be done. If the body be closely watched it will be found that it responds in its capacity of work to its food supply, as accurately and delicately as does the steam engine to its fuel supply. Without fuel, and the proper supply of fuel, force cannot be got from the engine, and without food and its proper supply in amount and quality, work cannot be got from the human creature. A person may indeed subsist on very low diet, or even exist in a quiescent state for some time without food at all, if supplied with water; but a life of health and vigour can only be maintained on the condition that the body is properly fed and nourished. To ascertain what is the proper amount of nourishment is my object.

**Subsistence Diet.**—We learn from studying prison dietaries, the feeding of famine-stricken populations, and the diet of our own poor in London what is the smallest amount of food on which the body can live, but not do hard work. This is three pounds of meat, with a pound of fat on it, or the same quantity of butter or lard, two quartern loaves, and about an ounce of salt a week. For meat, if unattainable, can be substituted two extra quartern loaves, or about a stone and a half of potatoes, or between 5lb. and 6 lb. of oatmeal. Thus, we see that a person can actually exist on four quartern loaves and a pound of butter or lard a week without being gradually starved. This is, however, the diet of bare existence; on it a person can do no work bodily or mental, or he will certainly break down. Children, it must be remembered, in whom tissue change is rapid and growth is taking place, require more than a subsistence diet.

**A Working Diet.**—Work may be divided into three degrees: 1. Moderate, which may be represented by a daily walk of from five to seven miles. Such is the amount of work done by soldiers on home service, by clerks, or the ordinary person in easy circumstances. Judging from army dietaries, five lbs. of meat and seven lbs. of bread weekly, with the addition of vegetables, milk, and vegetables are sufficient. 2. Active work, such as is accomplished by soldiers in campaign, letter-carriers, artizans, and which can be represented by a walk of twenty miles. These require a fifth more nitrogenous food and added starchy and fatty foods. 3. Hard work means the work got through by navvies, miners, &c. These as a rule eat increased quantities of meat if they can afford to do so. Science teaches, however, that in this they err, and that the force for hard work can be got at much less expense to the purse, and more easily by the body, from certain vegetables and from starchy and fatty foods than from large quantities of meat. An exclusively meat diet is wasteful as well as costly.

Now let us consider these dietaries and bold statements of fact in the light of experience, and learn something of how the poor live. In a thoughtful article by Mrs. S. A. Barnett (which was published first in the *National Review* for July, 1886, and republished in a book entitled "Practicable Socialism," a series of papers by herself and her husband), the application of correct scientific principles of diet to the needs and possibilities of the poor is considered. The article should be carefully studied by all who feel themselves called upon to decide social questions either theoretically or practically. Very much has been written about dietaries, and the amount of food necessary to raise so many foot-tons, in other words, to enable a navvy or docker to do his daily tasks, but no one, except Mrs. Barnett, has, so far as I know, taken the trouble to turn the percentages and quantities of carbonaceous and nitrogenous food required to maintain health, into economical dishes of potatoes and meat for a family of a working man and his wife and eight children and to show us the cost, of living to a farthing.

Assuming that, on the lowest estimates, a working man requires 16 oz. of carbonaceous and 4 oz. of nitrogenous food a day; his wife 12 oz. of carbonaceous and 3 oz. of nitrogenous food; and his eight children an average of 8 oz. of carbonaceous, and 2 oz. of nitrogenous food a day each; the total indicates that 92 oz. of carbonaceous and 23 oz. of nitrogenous food have to be daily provided. To show how this can be done with all the advantages of scientific culinary knowledge, Mrs. Barnett gives the following daily *ménus*, which I make no excuse for quoting at length:—

| Quanity of Food. | Cost. s. | d. | Carbonaceous. oz. | Nitrogenous. oz. |
|---|---|---|---|---|
| **Breakfast—Oatmeal Porridge.** | | | | |
| 1¼ lb. of Oatmeal .. | 0 | 2½ | 14 | 3 |
| 1½ pint Tinned Milk | 0 | 1½ | 2¼ | 1 |
| ½ lb. Treacle ...... | 0 | 1½ | 7 | — |
| **Dinner—Irish Stew.** | | | | |
| 1¼ lb. Meat ...... | 0 | 8 | 3½ | 3½ |
| 4 lb. Potatoes .... | 0 | 2½ | 14 | 2 |
| 1¼ lb. Onions .... | 0 | 1 | 5½ | 1¼ |
| A few Carrots .... | 0 | 1 | ¼ | — |
| ½ lb. Rice ........ | 0 | 1 | 7 | ½ |
| 1½ lb. Bread ...... | 0 | 2¼ | 13½ | 2¼ |
| **Tea—Bread and Coffee.** | | | | |
| 2½ lb. Bread ...... | 0 | 3¾ | 22½ | 3¾ |
| 2½ oz. Coffee ...... | 0 | 2½ | ¼ | ¼ |
| 1½ pint Tinned Milk | 0 | 1½ | 2¼ | 1 |
| Total ...... | 2 | 5 | 92 | 18½ |

Here there is a short allowance of nitrogenous food, which would be corrected by a rather larger allowance of bread. I will give another of these thoughtful daily *ménus*:—

| Quantity of Food. | Cost. s. | d. | Carbonaceous. oz. | Nitrogenous. oz. |
|---|---|---|---|---|
| **Breakfast—Bread and Cocoa.** | | | | |
| 2½ lb. of Bread.... | 0 | 3¾ | 22½ | 3¾ |
| 1½ oz. Cocoa ...... | 0 | 1½ | ¾ | ¼ |
| 1 pint Tinned Milk | 0 | 1 | 1¼ | ½ |
| 2 oz. Sugar ...... | 0 | ½ | 1½ | — |
| **Dinner—Lentil Soup, Toasted Cheese.** | | | | |
| 1½ lb. Lentils...... | 0 | 3 | 15 | 6 |
| 1 lb. Cheese ...... | 0 | 8 | 4½ | 5½ |
| 1½ lb. Bread ...... | 0 | 2¼ | 13½ | 2¼ |
| **Tea—Rice Pudding and Bread.** | | | | |
| ¾ lb. Rice ........ | 0 | 1½ | 10½ | ¾ |
| ½ pint Tinned Milk | 0 | 1½ | 2¼ | 1 |
| 2 oz. Sugar ...... | 0 | ¼ | 1½ | — |
| 1½ lb. Bread ...... | 0 | 2¼ | 13½ | 2¼ |
| Total ...... | 2 | 1¼ | 86½ | 22¼ |

It will be noted that the family is strictly teetotal, and that no extras of any kind can be allowed.

**Wages and Starvation.**—Now, it is apparent that on the lowest estimate and with all possible care and knowledge, the daily necessary food of a working man, his wife, and eight children, cannot cost less than on an average 2s. 4d. a day, or 16s. 8d. a week. If he is in receipt of a regular wage of £1 a week, this leaves 3s. 4d. for rent, firing, clothing, and school fees. "It is not to be done!" exclaims the impatient student. But it is done, and done by hundreds of thousands, and happy is the family who can depend upon a regular wage of £1 a week. How is it done, however? By starving. In Mrs. Barnett's pathetic words, "The children have to put up with less than they need; the mother goes without rather than let the children suffer, and thus the new baby is born weakly and half nourished, the children develop greediness in their never-satisfied and but partly-fed frames, and the father, too often insufficiently sustained, seeks alcohol, which, anyhow, seems to pick him up and hold him together; though his teetotal mates assure him it is only a delusion." Mrs. Barnett sums up the whole matter in these words, "While wages are at the present rate the large mass of our people cannot get enough food to maintain them in robust health." The results are scrofula, consumption, skin diseases, the exhausting diseases of the bones from which the children of the poor suffer; the want of power to recover from acute diseases; it is the poor and ill-fed that the epidemics of cholera and influenza sweep to their graves—a stunted and physically degenerate population. The moral and mental results are that ill-nourished brains are incapable of sustained intellectual effort, or even of correct and consecutive thinking; and hence that a degenerate morality and low cunning takes the place of a robust conscience and trained intelligence, and it is partly thus the "criminal classes" of our latter-day civilization are produced. Mrs. Barnett, with her intimate knowledge of the lives of the poor of London, among whom she has lived for the past twenty years, also shows how in the desperate struggle to obtain even an insufficient supply of food, for no funds are left the working man to provide books, for the means of culture, and the opportunities of social intercourse, which are as necessary for his mental health and development as food and drink are for his bodily welfare. Nothing is left, moreover, wherewith to purchase rest and peace by the seaside or in the country, and nothing to meet the severe tax of sickness or convalescence.

How this state of things is to be cured taxes the mind of the philanthropist, the economist, and the socialist; that it is intolerable there is no doubt. We have long been accustomed to boast of our wealth, and to be proud of our national resources; but the squandering of the rich, which is apparent to all, blinds our eyes to the wants of the poor, which are hidden. We forget, moreover, in estimating the national wealth that the prosperity of a nation must not be estimated by the spending power of the rich, but by the purchasing power of the poor, and that as long as half our population cannot by any possible means obtain enough food to maintain health; disease, suffering, crime, and unrest will be the result.

